# Efficacy of blood flow restriction exercise during dialysis for end stage kidney disease patients: protocol of a randomised controlled trial

**DOI:** 10.1186/s12882-017-0713-4

**Published:** 2017-09-11

**Authors:** Matthew J. Clarkson, Steve F. Fraser, Paul N. Bennett, Lawrence P. McMahon, Catherine Brumby, Stuart A. Warmington

**Affiliations:** 1 Institute for Physical Activity and Nutrition, School of Exercise and Nutrition Sciences, Deakin University, 221 Burwood Highway, Burwood, 3125 Australia; 2 Medical and Clinical Affairs, Satellite Healthcare, San Jose, CA USA; 3 School of Nursing and Midwifery, Deakin University, Burwood, VIC Australia; 4 Department of Renal Medicine, Eastern Health Clinical School, Melbourne, VIC Australia

**Keywords:** End-stage kidney disease, Dialysis, Exercise, Strength, Physical function, Blood flow restriction exercise

## Abstract

**Background:**

Exercise during haemodialysis improves strength and physical function. However, both patients and clinicians are time poor, and current exercise recommendations add an excessive time burden making exercise a rare addition to standard care. Hypothetically, blood flow restriction exercise performed during haemodialysis can provide greater value for time spent exercising, reducing this time burden while producing similar or greater outcomes. This study will explore the efficacy of blood flow restriction exercise for enhancing strength and physical function among haemodialysis patients.

**Methods:**

This is a randomised controlled trial design. A total of 75 participants will be recruited from haemodialysis clinics. Participants will be allocated to a blood flow restriction cycling group, traditional cycling group or usual care control group. Both exercising groups will complete 3 months of cycling exercise, performed intradialytically, three times per week. The blood flow restriction cycling group will complete two 10-min cycling bouts separated by a 20-min rest at a subjective effort of 15 on a 6 to 20 rating scale. This will be done with pressurised cuffs fitted proximally on the active limbs during exercise at 50% of a pre-determined limb occlusion pressure. The traditional cycling group will perform a continuous 20-min bout of exercise at a subjective effort of 12 on the same subjective effort scale. These workloads and volumes are equivalent and allow for comparison of a common blood flow restriction aerobic exercise prescription and a traditional aerobic exercise prescription. The primary outcome measures are lower limb strength, assessed by a three repetition maximum leg extension test, as well as objective measures of physical function: six-minute walk test, 30-s sit to stand, and timed up and go. Secondary outcome measures include thigh muscle cross sectional area, body composition, routine pathology, quality of life, and physical activity engagement.

**Discussion:**

This study will determine the efficacy of blood flow restriction exercise among dialysis patients for improving key physiological outcomes that impact independence and quality of life, with reduced burden on patients. This may have broader implications for other clinical populations with similarly declining muscle health and physical function, and those contraindicated to higher intensities of exercise.

**Trial registration:**

Australian and New Zealand Clinical Trial Register: ACTRN12616000121460.

## Background

End-stage kidney disease (ESKD) is the 5th stage of chronic kidney disease, characterised by a failure of the kidneys to adequately filter blood [1]. ESKD is estimated to affect as many as 2 million people worldwide, approximately half of whom do not receive adequate treatment [2, 3]. Treatment requires either transplantation, or if transplantation is unavailable patients are solely reliant on regular dialysis [4]. Largely due to the regularity of dialysis as a treatment, ESKD is among the most common reasons for hospitalisation [2]. However, despite dialysis being considered one of the most efficacious means of treating ESKD, it does not guarantee that life can be sustained, with 12 month mortality rate following initiation of dialysis estimated to be 10–12% [5].

ESKD patients suffer from anaemia, fatigue, muscle atrophy, decreased aerobic capacity, reduced participation in physical activity, increased risk of falls, and decreased quality of life [4, 6–8]. Patients with ESKD also demonstrate significant decline in physical function and tasks reflective of activities of daily living [9–13]. This diminished physical function leads to a significant loss of independence with 95% of people with ESKD not fully independent and in need of assistance with at least one activity of daily living (ADL) [14]. This is more significant considering that an inability to independently complete ADLs is a major predictor of mortality for ESKD [14]. Exercise is well tolerated among patients with ESKD and is an efficacious method for ameliorating these adverse symptoms characteristic of ESKD [15–18].

Heterogenous methodology, prescription and reporting among ESKD exercise studies make it difficult to draft guidelines on the dosage and mode of exercise training to derive the best outcomes for ESKD patients [8, 19]. The current guidelines for exercise training with ESKD recommend it encompasses aerobic (4 sessions; 45 mins), resistance (2 sessions; 20 mins), and flexibility (7 sessions; 10 mins) exercise [20]. The cumulative time commitment for these recommendations is substantial, and would require training on both dialysis (during first 2 h of dialysis) and non-dialysis days [20]. However, most randomised controlled trials examining exercise regimens in ESKD have only utilised aerobic training, most commonly stationary cycling [20]. Aerobic exercise training alone does not traditionally increase strength and hypertrophy, nor produce significant improvements in physical function, all of which are needed among this population [21].

Adherence to exercise training programs, regardless of aerobic, resistance or combined modalities, is also poor among ESKD patients, with more than 20% choosing not to participate, citing issues such as lack of time, lack of energy, too much trouble, and resistance training being too difficult [22]. Of those that chose to participate in exercise training, the dropout rate was more than 25% for programs utilising exercise training on non-dialysis days [22]. While there is still an increased dropout rate among patients with ESKD when exercise is completed intradialytically (approximately 17%) [22], it is lower than that seen on non-dialysis days. This suggests that intradialytic exercise is still preferable with regards to participant retention than exercise on non-dialysis days.

Resistance training poses further complications in the setting of a dialysis unit as the required equipment is cumbersome, and poses a high risk for cross-infection. Less cumbersome washable elastic resistance bands may be one option [23]. Exercise training during dialysis also needs to be completed in the first 2 h of dialysis sessions [24]. Therefore, finding a solution that may provide improvements in all key outcomes as efficiently as possible, allowing for completion during dialysis would be preferable.

Blood flow restriction exercise training uses pressurised cuffs fitted proximally on the active limbs during exercise training, and can elicit the above benefits at lower exercise intensities than non- blood flow restriction training [25, 26]. Blood flow restriction may be one method for achieving outcomes reflective of both resistance, and aerobic exercise training while providing an efficient, simple exercise prescription model in the dialysis setting. Blood flow restriction aerobic exercise training has been shown to increase muscle size and strength more than equivalent-intensity non-blood flow restriction aerobic exercise training, while still providing comparable or greater improvements in cardiovascular fitness [25, 27, 28].

While blood flow restriction exercise training is used predominately in single joint isolation resistance training exercises such as knee extension, it has also been applied during aerobic exercise training such as walking or cycling [25, 29–32]. Common blood flow restriction aerobic exercise training protocols utilise short exercise durations of 10 to 20 min [25, 32–34]. Such studies utilising blood flow restriction with aerobic exercise training have demonstrated increases in lower limb muscle volume and strength of up to 8% and 16%, respectively [25, 32, 33]. However, it should be noted that without blood flow restriction, low-intensity aerobic exercise training is generally insufficient to increase muscle volume or strength, unless there is previous muscle atrophy or the initial level of participant physical activity is extremely low [35]. Even in such instances, improvements in muscle volume and strength are unlikely to be of similar magnitude as seen with blood flow restriction [35].

Regardless of the presence of blood flow restriction, aerobic exercise training improves aerobic fitness provided that the exercise is of sufficient intensity [36]. Indeed, studies have demonstrated that low-to-moderate intensity blood flow restriction aerobic exercise training is able to increase aerobic capacity when exercising at intensities equivalent to 40% of peak exercise oxygen consumption (⩒O_2_peak) [32, 33, 37].

Studies examining the haemodynamic responses to blood flow restriction identify blood flow restriction aerobic exercise training as having lower haemodynamic stress than high intensity resistance training, while providing similar outcomes [38–40]. As such, blood flow restriction is purported to be a potentially beneficial addition to exercise for clinical populations who may be contraindicated to high-intensity resistance training. While blood flow restriction exercise is a novel addition to exercise among ESKD patients, pilot data from this laboratory (unpublished) indicates that haemodynamic responses (systolic blood pressure, diastolic blood pressure, mean arterial pressure, heart rate) do not differ markedly between low-intensity blood flow restriction and non-blood flow restriction aerobic exercise both performed during haemodialysis in this population.

Aerobic capacity and muscle strength are both key physiological factors underpinning physical function [41] and both can improve with blood flow restriction aerobic exercise training. As declines in physical function, muscle strength, and aerobic capacity, as well as accelerated muscle atrophy are common among patients with ESKD, blood flow restriction aerobic exercise training may present an opportunity to increase the efficiency of time spent exercising for this population.

This study will examine the efficacy of blood flow restriction aerobic training among patients with ESKD on haemodialysis (HD). We hypothesise that blood flow restriction aerobic exercise training will improve physical function, muscle strength, and muscle size among patients with ESKD beyond that of traditional aerobic exercise training.

## Methods

### Design

**Fig. 1:**
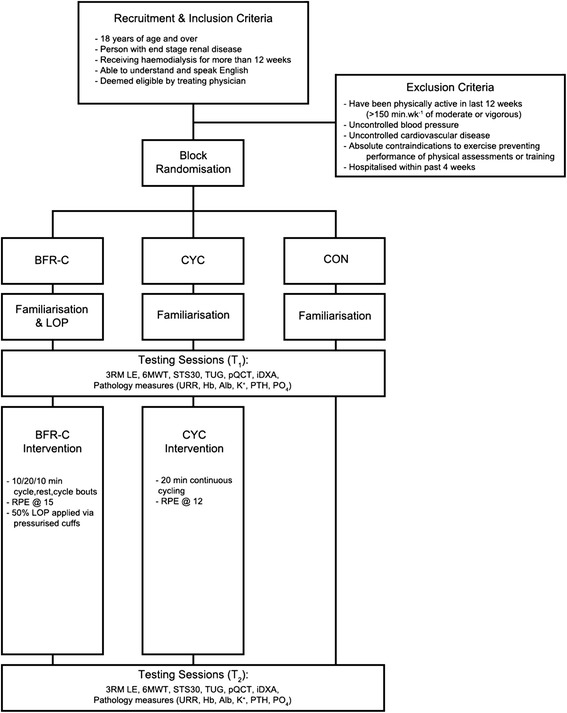
Study Flow Chart. BFR-C – Blood flow restriciton cycling group; CYC – Traditional cycling group; CON – Usual care control group; LOP – Limb occlusion pressure; 3RM LE – Three-repetition Maximum Leg Extension; 6MWT – 6 min walk test; STS30–30-s sit-to-stand test; TUG – Timed up and go; pQCT – Peripheral Quantitative Computed Tomography; iDXA – Dual X-ray Absorptiometry; URR – Urea Reduction Ratio; Hb – Haemoglobin; Alb – Albumin; K^+^ − Potassium; PTH – Parathyroid Hormone; PO4 – Phosphate; RPE – Rating of perceived exertion

This is a 12-week randomised controlled trial consisting of three groups randomised to exercise or usual care (Fig. 1). Participants will be randomised to one of three groups: a blood flow restriction cycling exercise training group (BFR-C), a non-blood flow restriction cycling exercise training group (CYC), or a non-exercising, usual care control group (CON).

### Sample size calculation

The most relevant outcome for blood flow restriction training studies is knee extensor muscle strength, and this was used as the main primary outcome measure for calculating required sample size. Based on data from previous blood flow restriction research [25, 29, 32, 33], and previous exercise for ESKD research [18, 42, 43], the projected percentage changes in knee extensor muscle strength for each of the three groups are CON = −3%, CYC = 2%, BFR-C = 12%. The common standard deviation within group, based on the prior research was determined to be 15. Thus, to achieve a power of 80% at an α level of 0.05, 20 participants per group will be required to detect a difference between the expected means for percentage strength improvement. However, based on previous work conducted by this research group a conservative attrition rate of 20% is expected over 3 months due to voluntary patient withdrawal, death, additional hospitalisation, transfer to another facility, or patients receiving transplants [23]. Thus, a total of 75 participants will be recruited and randomly allocated to one of the three groups.

### Recruitment and screening of participants

Participants will be recruited through recommendation and referral from treating physicians at a number of dialysis clinics linked to a major public hospital service in a large metropolitan Australian city. Additionally, nurse unit manager recommendations and fliers briefly outlining the study posted within the dialysis clinics will be used to help identify prospective participants. Prospective participants will be screened initially face-to-face or via telephone by a member of the research team. Following this, confirmation from the treating physician will be obtained if not implied by referral. Participants will be required to provide written, informed consent prior to participation in the study.

### Inclusion and exclusion criteria

Eligible participants will be male and female stable chronic (>3 months) haemodialysis patients aged between 18 and 80 years. Participants will be deemed medically eligible by their treating physician before participation in the present study. Participants will be excluded if they do not understand English and are unable to complete or comprehend the surveys or study documents; if within the previous 12 weeks they have participated in regular physical activity or sport (>150 min.wk.^−1^) of moderate or greater intensity [44], or structured resistance training (> 1 session.wk.^−1^); if they have symptomatic peripheral vascular disease, limb ischemia, untreated symptomatic cardiovascular disease, or any other absolute contraindications to exercise training (such as musculoskeletal factors or neurological conditions) that may affect their ability to perform physical assessments or exercise training protocols in the present study; if they are currently smokers; or if they are pregnant or have required hospitalisation for non-dialysis reasons in the 4 weeks prior to the study’s commencement. Patients will also be deemed unable to exercise during individual dialysis sessions if they present with fluid overload (> 5% above dialysis base weight) as this can cause reduced cardiovascular reserve [45]. Similarly, if patients have systolic blood pressure > 180 mmHg, or diastolic blood pressure < 90 mmHg prior to exercise they will be deemed unsuitable for exercise on that day (excluding the first blood pressure reading before dialysis, which is known to be unreliable) [46]. Of clinical concern is the known propensity for HD patients to become hypotensive (potentially more so post-exercise) during dialysis [47]. Although this is uncommon and our pilot data indicates this is not exacerbated by blood flow restriction, to ensure the safety of all patients they will be monitored for chest pain/discomfort, dyspnoea, lower limb pain, symptoms of severe hyper- or hypotension, and other signs of adverse events, which will be reported to the ethics committee of both the treating hospital and Deakin University.

### Randomisation

Randomisation will be conducted at a participant level prior to familiarisation and baseline testing, and will randomise participants by blocks of 4 via a computer-generated random number sequence by an independent researcher. Blinding of intervention group to clinical staff and participants will not be possible once the intervention commences.

### Exercise intervention

Exercise training sessions will occur intradialytically for all exercising participants and will be completed within the first 2 h of the session to avoid loss of exercise training quality [24, 48]. All sessions will be supervised by a member of the research team. Exercise training sessions will consist of cycle exercise for both BFR-C and CYC groups. Both BFR-C and CYC groups will complete a total volume of 20 min cycling at a relative intensity for each participant to be dictated by rating of perceived exertion (RPE), similar to previous exercise and ESKD studies [17, 49, 50]. This will also be compared to measurements of percentage age-predicted maximum heart rate (APHR_max_) in order to determine if targeted RPE needs to be adjusted to account for potential discrepancies between patient subjective intensity and objectively observed intensity. If participants are unable to maintain the prescribed cadence at this workload for the full duration, they will be instructed to complete as much of each cycling bout as possible.

#### Age-predicted maximum heart rate

APHR_max_ will be used as a relative indicator of intensity during exercise training sessions. Approximately 50–70% of participant’s age-predicted APHR_max_ will be used as an objective target to indicate whether participants in BFR-C or CYC are exercising at the prescribed intensity for this study. This will be achieved using a fingertip heart rate monitor and pulse oximeter (CMS50, Contec Medical Systems Co., Ltd., Qinhuangdao, Hebei Province, China) placed on the second or third finger of the participant’s hand on the contralateral limb to their arteriovenous fistula. This will be measured and recorded during the final minute of each period of active cycling within a session to correspond with measures of RPE.

#### Blood flow restriction cycling group

The BFR-C group will complete an intermittent protocol of 10 min cycling, 20 min rest, 10 min cycling. During these sessions, participants in the BFR-C group will have a blood flow restriction cuff fitted to the proximal end of each thigh, which will be inflated continuously at 50% of a pre-determined limb occlusion pressure (LOP) throughout the full duration of cycling. Participants will be required to cycle at an RPE of 15 (‘Hard’ on the Borg 6 to 20 scale [51]), expected to be equivalent to at least 60% of APHR_max_ for each 10 min cycling period. The prescribed volume and intensity represents a balance between prior blood flow restriction protocols and those utilised in the aerobic training components of HD studies, while accounting for potentially very low levels of physical function among the participants [6, 8, 52].

#### Blood flow restriction procedure

Blood flow restriction will be applied using an automatic tourniquet system (A.T.S 3000, Zimmer Inc., OH, USA). The ATS produces a defined, controlled pressure via adjustable pneumatic cuffs (52 cm long, 10.5 cm wide; bladder length 45 cm, bladder width 8 cm). The pressure is regulated to control for changes in muscle contractile pressures under the cuff in such a way that the occlusive pressure applied to the limb is constant.

The cuffs will be applied to the proximal aspect of the thigh prior to commencing exercise, and gradually inflated, as detailed by Abe et al. (2006) [25]. Blood flow restriction pressure will be set at 50% of pre-exercise resting LOP. LOP will be calculated during a familiarisation session for participants allocated to the BFR-C group using a digital plethysmograph (Pulse Sensor, Zimmer ATS 3000) applied to the second toe [40, 53]. A seated or recumbent posture will be used for LOP as it most closely matches the posture that will be used during the exercise training sessions. To determine LOP, the pneumatic cuffs are inflated to the point at which the plethysmograph can no longer detect blood flow to the toe (blood flow completely occluded). This will be done for each leg and repeated to ensure accuracy (<20 mmHg), and an average of these measures will be taken. The 50% LOP that is to be applied during exercise training sessions does not fully occlude the limb in this manner, and allows for continuous blood flow during exercise.

#### Cycling group

The CYC group exercise training sessions will require 20 min continuous cycling at an equivalent intensity to the BFR-C group. However due to the absence of blood flow restriction, the equivalent intensity is often reported to have a lower RPE and mildly reduced heart rate response, regardless of exercise modality [38, 54, 55]. As such, the target RPE will be 12 (‘light to somewhat hard’ on the Borg 6 to 20 scale [51]) for the CYC group, which is expected to equate to at least 50% APHR_max_. This intensity is recommended for intradialytic aerobic exercise in the Exercise and Sport Science Australia (ESSA) position statement on exercise for chronic kidney disease [20]. This volume is reflective of protocols used by other studies examining exercise training among patients with ESKD, and represents a “normal” exercise training prescription for patients with ESKD [22, 56].

#### Progression

In order to ensure that training sessions maintain a sufficient intensity for all exercising participants, resistance applied to the pedals, cadence, or a combination of both variables will be adjusted on a session-by-session basis by a trained exercise physiologist such that the required RPE target (indicative of sufficient subjective intensity) is achieved. This accounts for any illness or excessive fatigue that may affect exertion during any given session. A rolling two-week average of RPE will be monitored (on the 6–20 Borg’s RPE scale [51]) to ensure progressive overload is being achieved over the course of the 3-month intervention.

#### Usual care control group

Participants allocated to the usual care control group will receive no additional access to exercise training and will receive minimal advice regarding exercise training throughout the study. Participants randomised to the usual care control group will be given access to an accredited exercise physiologist member of the research team, free of charge if they would like to discuss the benefits of commencing exercise training upon completion of the study.

### Data collection

Data collection will occur at baseline (T_1_) and 12 weeks (T_2_), with testing to be completed at the treating HD unit prior to the commencement of the participants’ HD session. Personal information, including relevant medical history will be collected prior to the initiation of testing or intervention upon receiving participant consent. This will include patient characteristics of age, gender, time in months that the participant has been receiving HD (dialysis vintage), basic anthropometric measures (height, weight, and BMI), relevant comorbidities and medications, transplant status, and type of dialysis access. Some or all of which may be taken, with approval, from the case file at the HD unit. The remaining data collected will include all measures of muscle function and physical function. Body composition scans will be performed on a non-dialysis day, separate from other testing procedures for participants who opt-in to this aspect of testing. Additional pathology measurements will include haemoglobin, albumin, potassium, parathyroid hormone, phosphate, urea reduction ratio (URR), creatine kinase and lactate. These measures will occur as part of routine pathology tests where possible, or will be requested out-of-cycle to coincide with the necessary period of the training intervention. Data will be coded and stored securely at Deakin University. Data collected from those who withdraw from the study will not be used for statistical analyses. This has been accounted for when completing the power analysis, with an additional 20% added to the target sample size. Attrition may be for a number of reasons, which will be ascertained upon withdrawal where possible.

### Primary outcome measures

#### Lower-limb muscle strength

Maximal lower limb muscle strength will be determined using three-repetition maximum (3RM) knee extension (HS-LE, Life Fitness, Victoria, Australia) [57]. A 3RM knee extension test measures the maximum weight that can be lifted for three consecutive repetitions of a knee extension exercise with good technique and full range of motion. The 3RM result will be input into a formula to derive equivalent one-repetition maximum for each participant [58].

#### Physical function

Objective physical function will be measured using the 30-s sit to stand test (STS30), the timed up and go test (TUG), and the six-minute walk test (6MWT).

The STS30 measures lower limb muscle strength and function and requires participants to stand from a chair and then return to the seated position as many times as possible in 30 s [59]. The test has also displayed strong comparative validity to lower extremity 1RM strength testing, particularly among community-dwelling older adults [59]. It has also demonstrated a high level of reliability among ESKD patients undergoing HD (ICC = 0.93) [60].

The TUG is a measure of dynamic balance and mobility requiring participants to stand from a chair, walk to and then around a cone placed 3 m away, walk back to the chair, and sit back down as quickly as possible. This test will be repeated three times, and an average time taken for accuracy purposes [61]. The TUG strongly reflects gait speed among older adults, and has shown strong test re-test reliability not only among older adults, but in ESKD patients undergoing HD (ICC = 0.91–0.96) [62–64].

The 6MWT is a self-paced assessment of aerobic capacity, and a strong index of all cause morbidity in older adults [65]. It requires participants to walk laps of a straight 30 m course with the objective to cover as much distance as possible in the six-minute duration [65]. The 6MWT is a reliable measure for patients with ESKD (ICC = 0.93–0.96) [60, 66]. It has also been validated against ⩒O_2_max for patients with ESKD, with moderate strength coefficients of correlation (*r* = 0.56–0.73) [11, 67].

### Secondary outcome measures

#### Muscle cross-sectional area and body composition

Total and regional body composition (lean mass, fat mass and percentage fat mass) will be assessed using iDXA (Lunar iDXA, GE Healthcare, Madison, WI). Muscle cross-sectional area around the femur diaphysis at 25% and 50% of femur length will be assessed using pQCT (XCT 3000, Stratec Medizintechnik, Baden-Württemberg, Germany), allowing for knee flexor and extensor musculature to be measured. Height and body mass will be assessed using a portable stadiometer and scales respectively.

#### Pathology measures

Pathology measurements will be taken during routine collections completed by the treating hospital. The exercise intervention will be timed such that these routine pathology measures occur within the 4 weeks prior to the commencement of the first training session (or prior to the 3 month usual care monitoring period for the CON). These additional measures will include haemoglobin, albumin, potassium, parathyroid hormone, and phosphate. URR will also be measured by the treating hospital as a part of patients’ quarterly pathology tests, providing it is within the 4 weeks prior to patients commencing their exercise intervention. If this cannot be achieved in a timely manner, an additional measure of URR will be requested for a single dialysis session prior to the commencement of the exercise intervention.

Two additional measures of URR, creatine kinase and lactate will also be required outside of the normal regimen for patients in either of the exercise training intervention groups, once during the first week of the exercise training intervention, and again during the final week of the exercise training intervention. An additional out-of-cycle measure of URR will also be required by participants in the usual care control group, unless this is scheduled to be completed within 2 weeks following their 3-month monitoring period for this study as a part of their quarterly routine pathology test. All measures are conducted at the beginning and upon completion of a single dialysis session to establish the difference in each marker over the course of that session. This will allow analysis of the impact of exercise and specific exercise intervention types (BFR-C and CYC) on dialysis adequacy, as well as providing objective proof that there is no major tissue damage as a result of the intervention.

#### Physical activity levels

The Community Healthy Activities Model Program for Seniors (CHAMPS) physical activity survey will be used to assess participation in a comprehensive list of low, moderate and vigorous physical activities [68]. This questionnaire will provide valuable information about changes in physical function and physical activity behaviour over the course of the study.

#### Symptom related quality of life

The POS-S Renal questionnaire will be given to patients during the familiarisation session to assess their quality of life in direct relation to the symptoms experienced as a part of their condition [69]. This questionnaire provides valuable feedback about any changes experienced by patients regarding quality of life and disease-specific symptoms as a result of the intervention.

### Statistical analysis

All statistical analyses will be performed using SPSS 22.0 (IBM Corp, Chicago IL, United States of America). Participant characteristic and demographic data will be presented using descriptive statistics, and compared using independent t-tests for continuous variables and chi-square tests for categorical variables. The distribution of data will be assessed for normality with a Shapiro-Wilks test (*P* > 0.05). Non-normally distributed data will be compared using the Friedman non-parametric test. Otherwise, comparisons between groups for all continuous primary and secondary variables measured will be made using one-way analysis of variance (ANOVA). Main effects will be analysed using a Tukey post hoc test. A significance level of *P* < 0.05 will be adopted for all statistical tests. All data will be presented as means ± SEM unless stated.

## Discussion and conclusion

This study is the first to explore the efficacy of blood flow restriction aerobic exercise training among patients with ESKD. Additionally, it is one of the first to explore a battery of common clinical measures or physical function following a blood flow restriction aerobic exercise training program. The main objective of the study is to determine the effectiveness of blood flow restriction aerobic exercise training for improving the strength and physical function among patients with ESKD.

While exercise during dialysis is not a novel concept in the broader literature, the current exercise recommendations present a significant time and physical burden for patients with ESKD and does not have an established role in clinical practice. Therefore, this study is important given the well documented deficits in strength and physical function for patients with ESKD in conjunction with the rarity with which any exercise is adopted not only intradialytically, but in the broader ESKD population. It may also be valuable to include measures related to vascular stiffness (e.g. pulse wave velocity and vascular-related microRNAs), as this has also been shown to improve with exercise training, and may be enhanced with the addition of blood flow restriction [34, 70, 71].

It is expected that this study will provide an improved understanding of the role that blood flow restriction aerobic exercise training can play in providing an efficacious, time-efficient modality of intradialytic exercise for patients with ESKD, which caters to the population’s lower initial level of physical function and overall conditioning.

## Abbreviations

6MWT: 6-min walk test
ADL: Activity of daily living
APHR_max_: Age-predicted maximum heart rate
BFR-C: Blood flow restriction cycling group
CHAMPS: Community healthy activities model program for seniors questionnaire
CON: Usual care control group
CYC: Traditional cycling group
ESKD: End-stage kidney disease
HD: Haemodialysis
iDXA: Dual X-ray absorptiometry
LOP: Limb occlusion pressure
pQCT: Peripheral quantitative computed tomography
RPE: Rating of perceived exertion
STS30: 30-s sit to stand test
TUG: Timed up and go test
URR: Urea reduction ratio
⩒O_2_peak: Peak oxygen uptake
